# Directional Joint Bilateral Filter for Depth Images

**DOI:** 10.3390/s140711362

**Published:** 2014-06-26

**Authors:** Anh Vu Le, Seung-Won Jung, Chee Sun Won

**Affiliations:** 1 Department of Electronics and Electrical Engineering, Dongguk University-Seoul, 30 Pildong-ro 1-gil, Jung-gu, Seoul 100-715, Korea; E-Mails: levuanh.hut@gmail.com (A.V.L.); cswon@dongguk.edu (C.S.W.); 2 Department of Multimedia Engineering, Dongguk University-Seoul, 30 Pildong-ro 1-gil, Jung-gu, Seoul 100-715, Korea

**Keywords:** depth map, image filtering, joint bilateral filter, joint trilateral filter, Kinect

## Abstract

Depth maps taken by the low cost Kinect sensor are often noisy and incomplete. Thus, post-processing for obtaining reliable depth maps is necessary for advanced image and video applications such as object recognition and multi-view rendering. In this paper, we propose adaptive directional filters that fill the holes and suppress the noise in depth maps. Specifically, novel filters whose window shapes are adaptively adjusted based on the edge direction of the color image are presented. Experimental results show that our method yields higher quality filtered depth maps than other existing methods, especially at the edge boundaries.

## 1. Introduction

Recently, many researchers and developers around the world have shown great interest in the low cost Microsoft Kinect sensor for various depth-based applications such as games and human-machine interactions [1,2]. A software toolkit for the Kinect provided by Microsoft [3] has been frequently updated to support researchers in expanding the scope of applications. However, the depth sensing mechanism of the Kinect produces incomplete depth data, yielding unavoidable noise and holes in the depth map. These defects restrict the Kinect from use in more depth-dependent applications. Thus, a filtering process for improving the quality of depth maps is essential for advanced Kinect applications. In the recent survey [4], it has been shown that if the noise and holes in the depth map of the Kinect are removed by filtering, the performance of depth-based gesture recognition and body tracking algorithms can be improved significantly.

A number of methods have been proposed for filling the holes in the depth map, which are mostly based on a pre-calibrated color image as well as the noisy depth map. In [5], a directional Gaussian filter is used to fill the holes by taking the edge information into account. The idea of directional filtering is promising, but the guidance information from edge direction in the hole area is not accurate enough compared with the information from edge direction of the corresponding color image. The hole-filling by a fixed-size window without considering the surrounding region of the hole may be the cause of the limited performance in [5]. To utilize the co-aligned color image as well as the depth map the joint bilateral filter (JBF) [6] and the guided filter [7] were proposed. Although these methods [6,7] can reduce the blur effect at the edge region, the blurring effect still remains when there is no significant intensity difference around depth discontinuities. The recent approach described in [8] uses a complicated foreground/background pixel classification method [9] in the temporal domain and applies different JBF filter kernels to the classified pixels. Although the method [8] produces temporally smooth depth maps, it still suffers from the drawback of [6,7] and improves only the foreground objects. The problem of hole filling is also considered in [10,11] with the modification of the well-known fast marching-based image inpainting method [12]. In particular, the color structure is used to determine the weighting function for the hole filling. The methods in [10,11], however, produce low quality depth maps if original depth and corresponding color images are not well aligned. A modified method of the joint-trilateral filter (JTF) [13] which uses both depth and color pixels to estimate the filter kernels is used to improve the quality of both depth and color images. This method assumes that the depth map has been processed such that there are no hole pixels and the depth map has enough quality to be used with the color image to determine the filter kernel, which requires a high performance algorithm for depth map post-processing.

**Figure 1.**
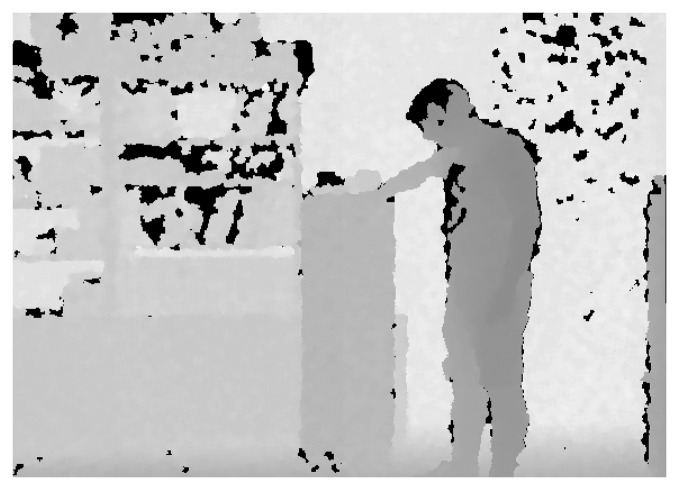
Example of imperfections in a Kinect depth map: Original depth map with hole pixels (represented as black pixels) detected by the “non-available” tags.

The Kinect depth sensor suffers from some imperfections resulting from various measurement errors caused by the distance to the sensor, the lighting conditions, occluded objects, and the object surfaces [14]. These measurement errors result in two types of defects in the depth map: noise (including random and blurring artifacts) and holes. Our approach to alleviate the imperfections of the Kinect depth map is to treat the noise and holes separately. Therefore, we first classify each pixel in the depth map into a hole pixel or a non-hole pixel. For this, the Kinect tags “non-available” for pixels with no returned signal and we classify these “non-available” pixels as hole pixels (see Figure 1). Besides, non-hole regions are usually noisy. Therefore, for the non-hole regions a bilateral filter with edge-dependent directional Gaussian kernels and a trilateral filter considering the similarity of depth pixels are selectively used according to the type of the pixels (edge or non-edge) to effectively remove the noise. Then, to fill the empty pixels in the hole regions, we propose a filter with adaptive local support (window), which consists only of the pixel values in the same region with the to-be-filled pixel. Specifically, the direction between the hole pixel and its closest edge pixel is used to determine the region to be used for the filtering. This direction and layout adaptive window help to sharpen depth edges at the depth boundaries while reducing the depth noise in the homogeneous (non-edge) regions.

In this work we propose the following novel approaches to make the depth map complete without holes and noise: (i) Depending on the existence of edge and hole, pixels in the depth map are classified into four groups, namely non-hole/non-edge, non-hole/edge, hole/non-edge, and hole/edge. For this, edges are determined from the color image and the holes are determined from the “non-available” tag of the Kinect depth map; (ii) Noise in the non-hole depth map are removed by JTF [13] and the blurring artifact in the object boundary of the non-hole depth map is removed by the proposed directional joint bilateral filter (DJBF); (iii) The filtered depth data of (ii) are used as a support window for the hole filling. Holes in the non-edge regions are filled by the proposed partial directional joint bilateral filter (PDJBF) and those in the edge regions are filled by the DJBF. Our selective filters for the classified depth regions are summarized in Table 1.

We note that the most closely related conventional method [8] also applies edge adaptive filters with reliable neighboring depth pixels. The proposed approach differs from the conventional method [8] in two major aspects. First, the filters used for the edge and non-edge pixels are different. The conventional method uses joint bilateral and median filters, whereas the proposed method uses trilateral and directional bilateral filters for the non-edge and edge pixels, respectively. The trilateral filter outperforms the joint bilateral filter in filtering noisy non-edge pixels, and the directional bilateral filter produces sharper depth edges compared to the median filter. We demonstrate the effectiveness of our choice of the filters through the experiments. Second, the conventional method uses foreground/background pixel classification to obtain reliable pixels to be used in hole filling. However, the classification is computationally demanding and the classification result is not trustworthy, especially when there are no significant intensity differences around depth discontinuities. Thus the conventional hole filling method tends to produce blurry depth edges. On the contrary, the proposed method adjusts the filter support in a way that only the neighboring pixels belonging to the same region of the hole pixel are used in the hole filling, resulting improved performance.

The rest of the paper is organized as follows: in Section 2, we describe the proposed adaptive filter kernel and window selection scheme. Section 3 provides our experimental results and comparisons with the existing methods. Section 4 concludes the paper.

## 2. Adaptive Directional Filters for Depth Image Filtering

**Figure 2.**
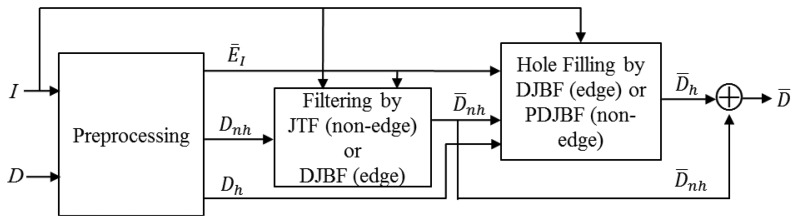
Block diagram for the proposed depth image filtering.

The Kinect depth sensor suffers from two types of imperfections: (i) noisy measurements of depth; (ii) holes of unmeasured depth. Our approach to enhance the imperfect depth image is to adopt separate filters for hole and non-hole regions. As shown in Figure 2, the depth image *D* is first classified into hole *D_h_* and non-hole *D_nh_* regions. Then, the filters are applied to the non-hole pixels to remove the depth noise, resulting *D̄_nh_*, and then the hole-filling scheme is used to fill the holes, resulting *D̄_h_*. The final depth *D̄* is the combination of *D̄_nh_* and *D̄_h_*. Since the color image *I* as well as the depth map *D* is available for the Kinect sensor, the filtering and hole-filling exploit the color image to locate the edge pixels in the depth map.

### 2.1. Preprocessing and Edge Detection

**Figure 3.**
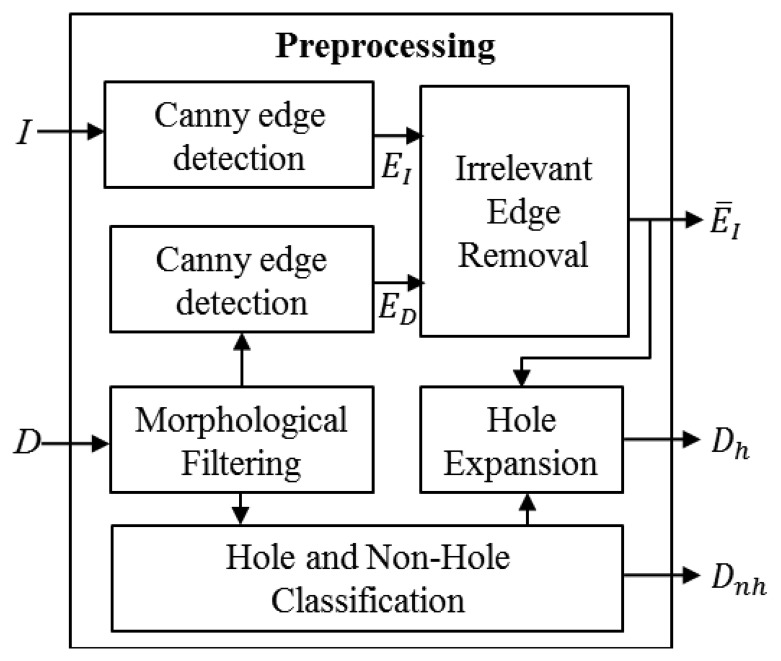
Edge detection and hole expansion in the preprocessing block of Figure 2.

As shown in Figure 3, the input depth map *D* is first pre-processed to remove small depth hole pixels that appear randomly between consecutive frames. To this end, morphological closing operation with 5 × 5 mask is applied to *D*, yielding the outlier-removed depth map. For the simplicity, let *D* hereafter denote the pre-processed depth map.

**Figure 4.**
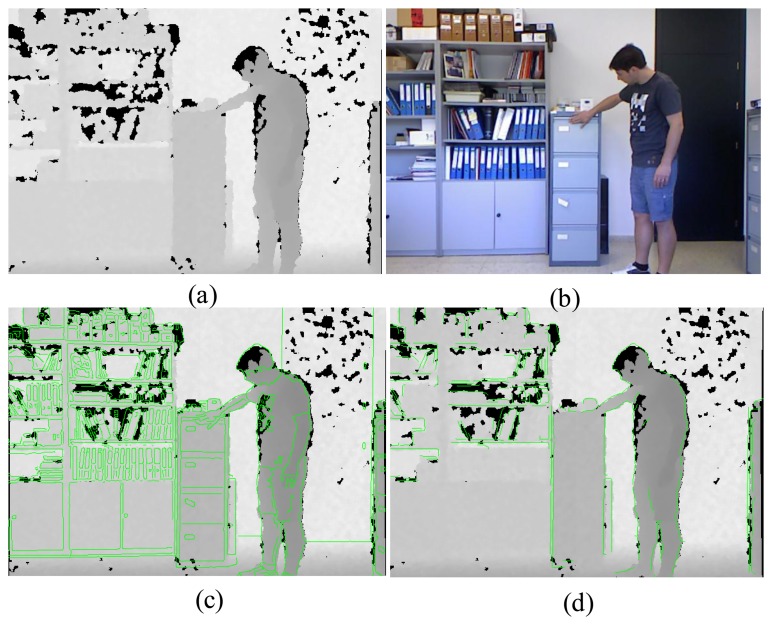
Noisy depth, color and overlay of depth and edge maps: (**a**) Noisy depth; (**b**) Color image; (**c**) Overlaid image by *D* and *E_I_*; (**d**) Overlaid image by *D* and *Ē_I_*.

The color image *I* from the Kinect is calibrated with the depth image such that the pixel positions in the depth image are nearly matched with the color pixel positions. Thus, the Canny edge detector is used to find the edge maps of the color and depth images, which are denoted as *E_I_* and *E_D_*, respectively. Then the edge pixels in *E_I_* that do not belong to the object boundary are removed to reduce the effect of textual details of the color image. To this end, for each color edge pixel in *E_I_*, if there exist no depth edge pixels inside a 7 × 7 window in *E_D_* centered at the corresponding depth position of the color edge, the color edge pixel is deemed as a misaligned pixel and removed from *E_I_*. After that, the edge pixels with the number of connected pixels lower than the threshold *T_n_* are also removed. Thus, the output edge map *Ē_I_* is expected to have edge pixels mostly around the object boundaries. Figure 4 illustrates an example of noisy depth and color images, and overlay of depth and edge maps.

In practice, the depth and color images cannot be calibrated perfectly. This makes the edge pixels of the color image be slightly misaligned with the corresponding edge pixels in the depth map. Therefore, the depth pixels around the misaligned edges in *Ē_I_* may not be correct, so in our method, all depth pixels inside a 7 × 7 window of each edge pixel *Ē_I_* are regarded as hole pixels, expanding the hole areas around the edge boundaries.

### 2.2. Filtering Non-Hole Regions

**Figure 5.**
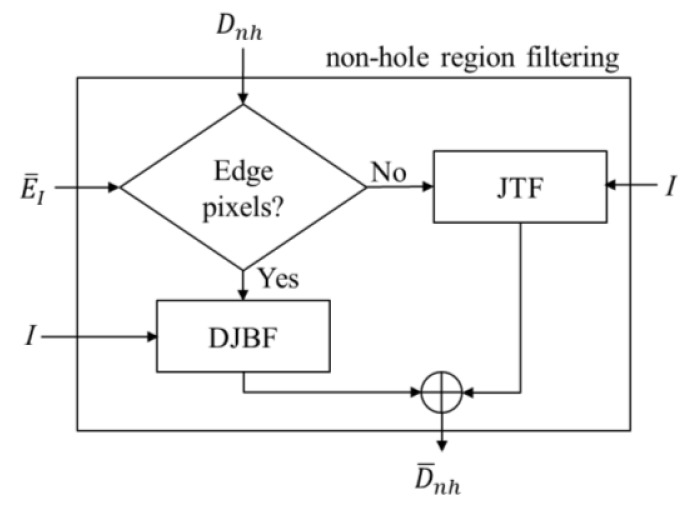
The flowchart of the non-hole region filtering.

Since the noise and the holes in the depth image are treated separately, we need to classify the depth image into non-hole (*D_nh_*) and hole (*D_h_*) areas. Note that the Kinect sensor provides tags of “non-available” for the pixels with no returned signals and we classify these “non-available” pixels as hole pixels. Figure 5 shows the flowchart of the non-hole region filtering.

The non-hole depth region *D_nh_* is usually noisy and the conventional JBF [6] can be considered for the noise filtering, which processes a pixel at position *p* = (*p_x_, p_y_*) using a set of neighbor pixels in the window Φ*^p^* of (2*w*+1) × (2*w*+1) as follows:

(1) $${\bar{D}}_{nh}=\sum_{q\in \Phi^{P}}D_{nh}(q)f_{s}^{d}(q_{x}-p_{x},q_{y}-p_{y})f_{r}^{c}(I^{p}-I^{q})$$

where Φ*^p^* = {*q* = (*q_x_, q_y_*)|*p_x_* − *w* ≤ *q_x_* ≤ *p_x_* + *w and p_y_* − *w* ≤ *q_y_* ≤ *p_y_* + *w*}. Also, *D_nh_*(*q*) and *D̄_nh_*(*p*) in Equation (1) represent the depth data at the pixels *p* and *q* in the non-hole regions. The spatial Gaussian kernel $f_{s}^{d}$ is defined as:

(2) $$f_{s}^{d}(q_{x}-p_{x},q_{y}-p_{y})=e^{-\frac{1}{2}\left(\frac{{\left(q_{x}-p_{x}\right)}^{2}+{\left(q_{y}-p_{y}\right)}^{2}}{{\text{σ}}_{s}^{2}}\right)}$$

The term $f_{r}^{c}$ measures the color difference between neighboring pixels as:

(3) $$f_{r}^{c}\left(I^{p}-I^{q}\right)=e^{-\frac{1}{2}{\left(\frac{I^{p}-I^{q}}{{\text{σ}}_{r}}\right)}_{2}}$$

Note that it has been shown that the JTF [13] performs better for the depth images. Therefore, instead of the classic JBF, we adopt the JTF [13] for the non-hole pixels. In particular, for the non-edge region in *Ē_I_*, we have:

(4) $${\bar{D}}_{nh}=\sum_{q\in \Phi^{P}}D_{nh}(q)f_{s}^{d}(q_{x}-p_{x},q_{y}-p_{y})f_{r}^{c}(I^{p}-I^{q})f_{r}^{d}\left(D_{nh}(p)-D_{nh}(q)\right)$$

In Equation (4) we consider the depth similarity around the neighborhood pixels as:

(5) $$f_{r}^{d}\left(D_{nh}(p)-D_{nh}(q)\right)=e^{-\frac{1}{2}{\left(\frac{D_{nh}(p)-D_{nh}(q)}{{\text{σ}}_{r}}\right)}^{2}}$$

This gives a higher weight to the pixel whose depth value is similar to that of the to-be-filtered pixel. However, for the edge region in *Ē_I_*, we propose to exploit the edge directions into the JBF of Equation (2) to preserve the edge sharpness. We have the DJBF as:

(6) $${\bar{D}}_{nh}=\sum_{q\in \Phi^{P}}D_{nh}(q)f_{ds}^{d}(q_{x}-p_{x},q_{y}-p_{y})f_{r}^{c}(I^{p}-I^{q})$$

where the directional Gaussian filter (DGF) is used for the spatial filter kernel as follows:

(7) $$\begin{array}{l} f_{ds}^{d}(q_{x}-p_{x},q_{y}-p_{y})=e^{-\frac{1}{2}\left(\frac{x_{\theta }^{2}}{{\text{σ}}_{x}^{2}}+\frac{y_{\theta }^{2}}{{\text{σ}}_{y}^{2}}\right)}\\ \begin{array}{rr} x_{\theta } & =(q_{x}-p_{x})\cos\theta -(q_{y}-p_{y})\sin\theta \\ y_{\theta } & =(q_{x}-p_{x})\sin\theta +(q_{y}-p_{y})\cos\theta \end{array} \end{array}$$

**Figure 6.**
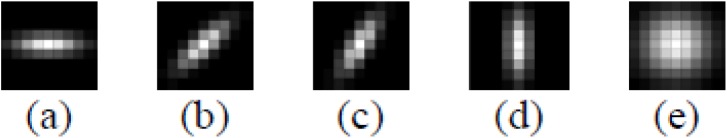
Example of 9 × 9 DGF kernels with σ*_x_* = 3, σ*_y_* = 1 in different angles: (**a**) 0°; (**b**) 45°; (**c**) 60°; (**d**) 90°; (**e**) the Gaussian filter with σ*_x_* = 3, σ*_y_* = 3.

Note that the depth data at the neighboring pixels of the non-hole pixel at the edge region in *Ē_I_* are unreliable because of the misalignment between the depth and color images. So, the purpose of using the DGF is to enhance the sharpness of the depth edges by giving a higher weight to the pixels along the edge direction of the to-be-filtered depth pixel. The DGF is rotated according to the edge direction *θ*, and its horizontal and vertical standard deviations σ*_x_* and σ*_y_* are controlled separately. Figure 6 illustrates the filter kernels of the DGF for different edge directions. The edge direction *θ* is given by:

(8) $$\theta =\tan^{-1}(g_{x}/g_{y})$$

where (*g_x_,g_y_*) denotes the spatial gradients in *x* and *y* directions.

### 2.3. Hole Filling

**Figure 7.**
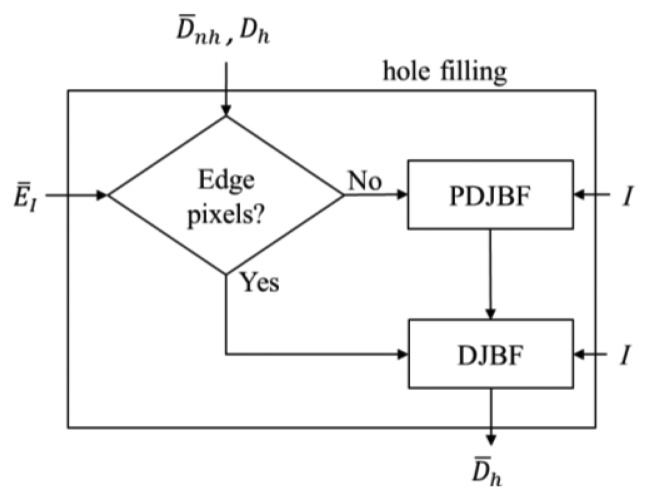
The flowchart of the hole region filling.

After filtering the depth pixels to obtain more confident depth values in the non-hole regions, those filtered depth data are used to fill the holes. First, to determine the origin of the holes, we exploit the edge information *Ē_I_* again to classify the holes into edge or non-edge regions. For the holes in the non-edge region we propose a partial directional joint bilateral filter (PDJBF) to fill the hole pixels, whereas the DJBF in Equation (6) is used to fill the hole pixels in the edge region. To enhance the performance of the hole filling at the edge regions, the hole pixels in the non-edge region are filled first. Figure 7 shows the flowchart of the proposed hole filling method.

**Figure 8.**
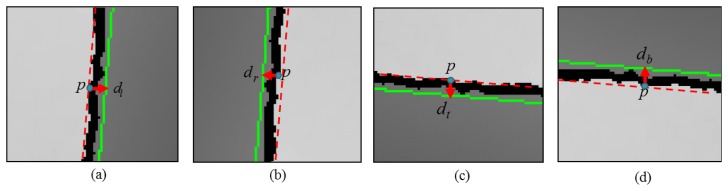
Illustration of hole pixels (black) and their nearby edges (green). Depending on the position of the nearby edges, four different directional hole filling operations are used: (**a**) left to right; (**b**) right to left; (**c**) top to bottom; (**d**) bottom to top.

Note that, in the Kinect depth maps, holes often appear at object boundaries. Thus, the directions of object boundaries need to be considered in hole filling. In our work, we take the four directional hole filling into account: filtering from left to right, right to left, top to bottom, or bottom to top direction. In particular, we determine which directional hole filling is required for each hole pixel by checking the nearest edge pixel and its edge direction. Hole filling from the left to right direction, for example, is performed when the nearest edge pixel in *Ē_I_* for the hole pixel is located on the right side as shown in Figure 8a. The green lines in this figure are the edges of *Ē_I_* overplayed with *D̄_nh_*. Note that the edge pixels are located at the object boundaries and they separate objects with different depth values. Therefore, it is important to find the object region that the hole pixels actually belong. In the case of Figure 8a, the hole pixels certainly belong to the left side region of the edge, hole filling from the left to right direction. The cases for the right to left, top to bottom, and bottom to top directions are also illustrated in Figure 8b–d. More specifically, to determine the origin of the regions for each hole pixel p, we need to calculate the distances to its nearest edge pixels in *Ē_I_* in the left, right, top, and bottom directions. The four distances, *d_l_, d_r_, d_t_* , and *d_b_*, are illustrated in Figure 8. Then, for instance, if the minimum distance turns out to be *d_l_*, then we decide that the hole pixel belongs to the left region of the edge and the directional hole filling is executed from left to right direction.

Once the direction of the hole filling is determined, to fill the hole pixels in the non-edge region, the proposed PDJBF uses the DGF as a spatial kernel, which can smooth images whilst retaining the edge details. The PDJBF is defined as follows:

(9) $$D_{f}^{p}=\sum_{q\in {\text{Ω}}^{p}}D_{m}^{q}f_{ds}^{d}(q_{x}-p_{x},q_{y}-p_{y})f_{r}^{c}(I^{p}-I^{q})$$

Note that the PDJBF is the same as the DJBF except for the filter support Ω*^p^*. Here the size of the filter support is adaptively determined to improve the performance of hole filling. Specifically, the window size is adjusted to use neighboring pixels that belong to the same region where the hole pixel belongs to. To this end, when the direction of the hole filling is from left to right, we find the minimum distance *d_w_, w* ∈ {*r,t,b*}. Then, the window size (2*w* + 1) × (2*w* + 1) is determined as:

(10) $$w=\{\begin{array}{ll} w_{\max} & if\;w_{\max}<d_{w}\\ d_{w} & if\;w_{\max}>d_{w} \end{array}$$

where *w*_max_ is a pre-fixed value. Similar manner is used to determine the window size for other hole filling directions.

**Figure 9.**
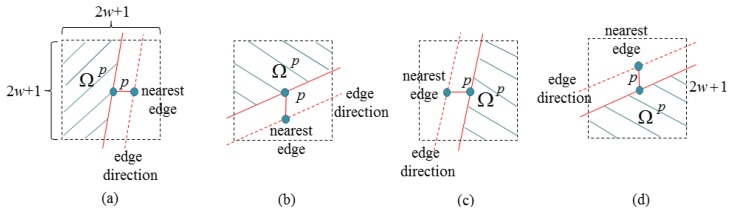
Filter range Ω*^p^* for (**a**) left to right; (**b**) top to bottom; (**c**) right to left; and (**d**) bottom to top directional hole filling.

Moreover, the pixels in the filter support Ω*^p^* of the PDJBF are selectively used according to the edge direction of the nearest edge pixel as shown in Figure 9 (only the pixels in the shaded region are used in filtering). For example, in case of the left to right hole filling as shown in Figure 9a, the pixels located at the left hand side with respect to the solid line are used in filtering. Similarly, we selectively use the pixels in the filter supports for other directions as shown in Figure 9b–d.

It is worth noting that the PDJBF is particularly effective in the hole filling for the regions consisting of depth discontinuities of several objects because our filter uses only the neighbor pixels that belong to the same object. After the hole filling of the non-edge region, the hole pixels at the edge region are filled using the DJBF. The DJBF used for the hole region filling is the same as the DJBF described in Section 2.2 except for the adaptive window size adjustment in Equation (10). The DJBF can enhance the sharpness of depth maps because the direction of the edge pixels in *Ē_I_* is taken into account to form the spatial kernel. After these processes, there exist small amount of unfilled hole pixels and these holes are simply filled by the JBF.

## 3. Experimental Results

In this section, we compare the results obtained with the proposed method to four conventional methods, namely the JBF [6], fast marching inpainting algorithm (FMI) [10], guided fast marching inpainting (GFMI) [11] without the guided image filter [7] (GFMI), and guided fast marching inpainting [11] with the guided image filter [7] (GFMIGF). The parameters, σ*_s_* = 3, σ*_r_* = 0.1, are the default values of [6] which compromise between the edge sharpening and smoothing effect. For the simplicity, we fix the parameters as recommended values [6]. Parameter values of σ*_x_* = 3, and σ*_y_* = 1 are also suggested by [5] to control the proportion between the long and short axes of the ellipse kernel. *w*_max_ set as 11 to compromise between the execution time and filtering performance. We empirically found that *T_n_* = 10 is sufficient to eliminate small cluttered edges.

**Figure 10.**
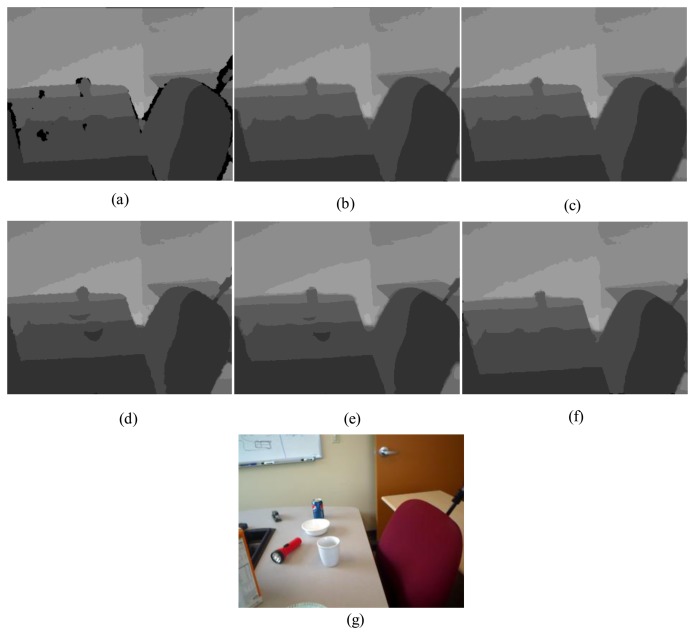
Experimental Results: (**a**) Original; (**b**) JBF; (**c**) FMI; (**d**) GFMI; (**e**) GFMIGF; (**f**) Proposed method; (**g**) Color image.

**Figure 11.**
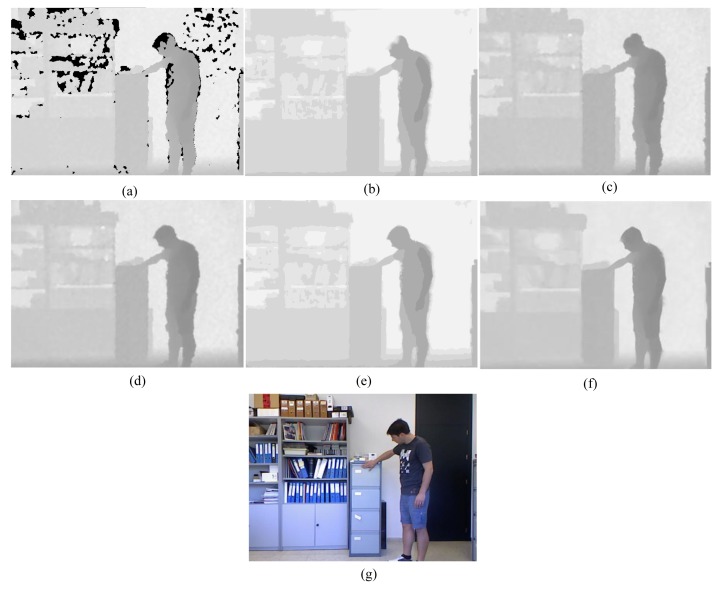
Experimental Results: (**a**) Original; (**b**) JBF; (**c**) FMI;(**d**) GFMI; (**e**) GFMIGF; (**f**) Proposed method; (**g**) Color image.

**Figure 12.**
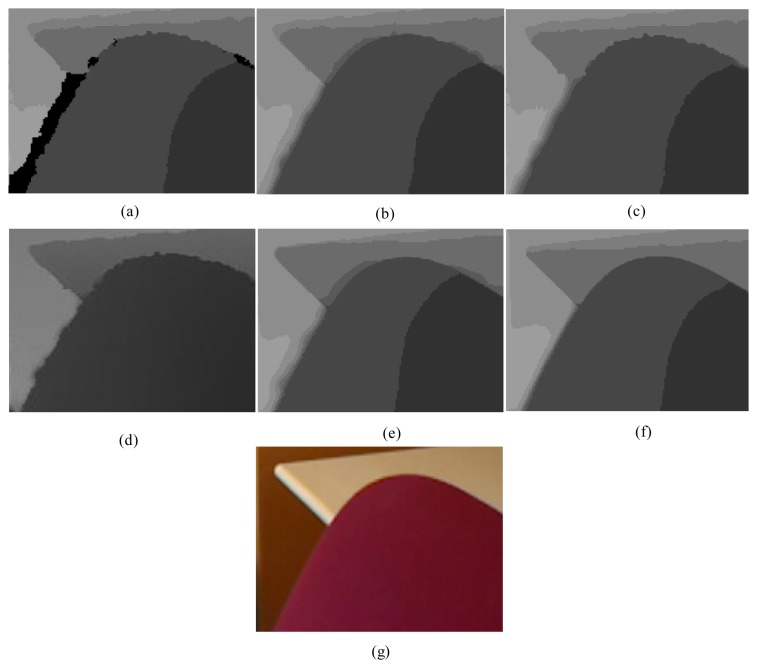
Zoomed-in results for Figure 10: (**a**) Original; (**b**) JBF;(**c**) FMI; (**d**) GFMI; (**e**) GFMIGF; (**f**) Proposed method; (**g**) Color image.

**Figure 13.**
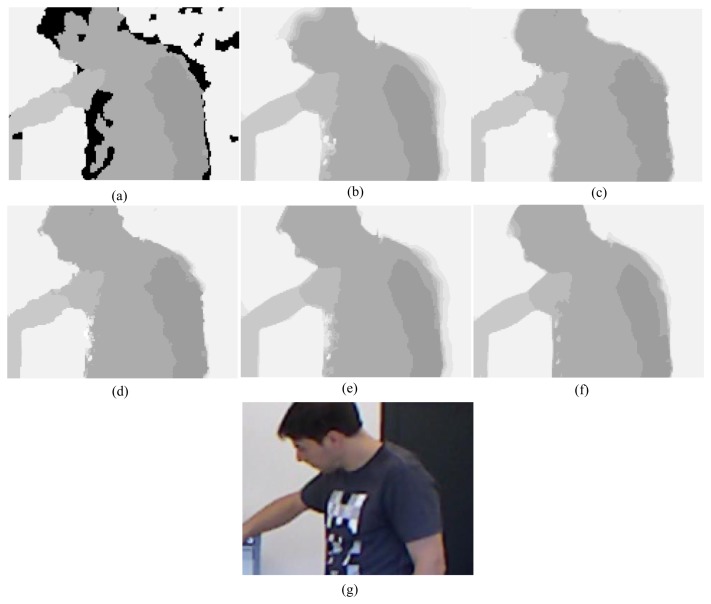
Zoomed-in results for Figure 11: (**a**) Original; (**b**) JBF; (**c**) FMI; (**d**) GFMI; (**e**) GFMIGF; (**f**) Proposed method; (**g**) Color image.

First, for the qualitative evaluation on the Kinect, the test images are obtained from two open Kinect databases [15,16]. The filtered Kinect depth maps of the proposed and conventional methods are shown in Figures 10 and 11. Figures 12 and 13 are their zoomed-in images for better subjective comparisons. As one can see, our method outperforms the conventional ones, especially at the pixels near object boundaries.

**Figure 14.**
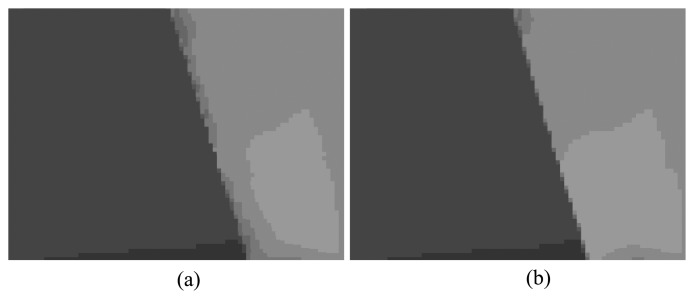
Visual comparison: (**a**) without the directional Gaussian kernel; (**b**) with the directional Gaussian kernel.

The power of the directional Gaussian term is demonstrated in Figure 14. As can be seen, without using the directional Gaussian term in Figure 14a, some aliasing artifacts appear at the filtered edges. On the other hand, these artifacts are removed if the directional term is included (see Figure 14b). In particular, when the directional term is not used, our filter does not significantly outperform the trilateral filter. This is because the directional term makes the filter supports of our DJBG and PDJBF include more neighboring pixels located along the edge direction of the filtered pixel.

**Figure 15.**
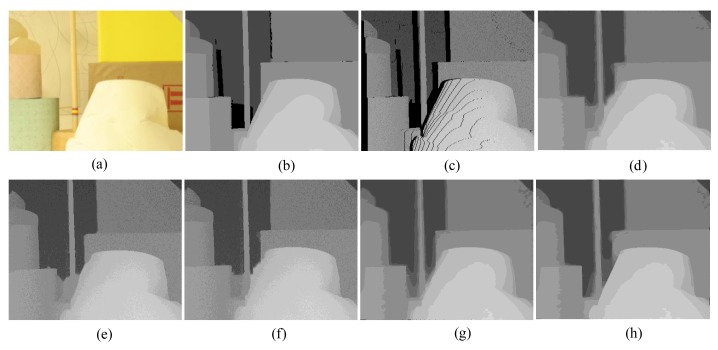
Results for Lampshade from the Middlebury database: (**a**) Color image; (**b**) Original depth map (right view); (**c**) Noisy image; (**d**) JBF; (**e**) FMI; (**f**) GFMI; (**g**) GFMIGF; (**h**) Proposed method.

**Figure 16.**
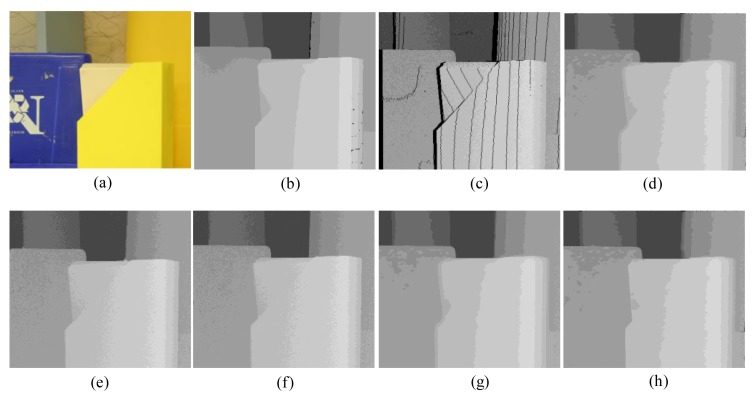
Results for Plastic from the Middlebury database: (**a**) Color image; (**b**) Original depth map (right view); (**c**) Noisy image; (**d**) JBF; (**e**) FMI; (**f**) GFMI; (**g**) GFMIGF; (**h**) Proposed method.

**Figure 17.**
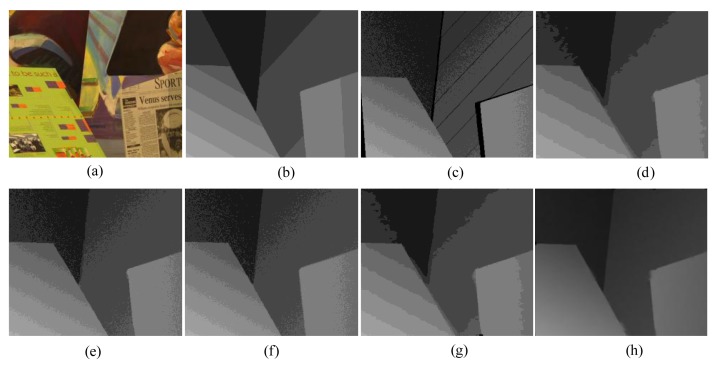
Results for Venus from the Middlebury database: (**a**) Color image; (**b**) Original depth map (right view); (**c**) Noisy image; (**d**) JBF; (**e**) FMI; (**f**) GFMI; (**g**) GFMIGF; (**h**) Proposed method.

**Figure 18.**
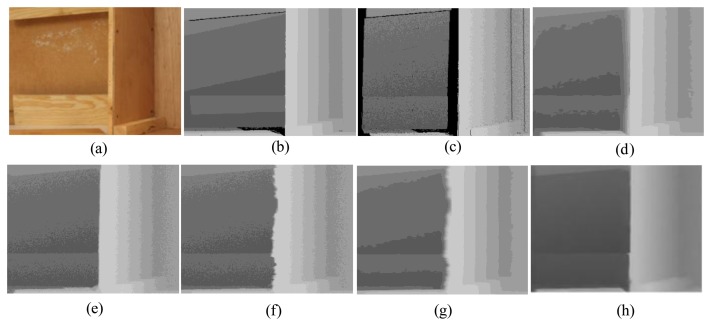
Results for Wood from the Middlebury database: (**a**) Color image; (**b**) Original depth map (right view); (**c**) Noisy image; (**d**) JBF; (**e**) FMI; (**f**) GFMI; (**g**) GFMIGF; (**h**) Proposed method.

**Figure 19.**
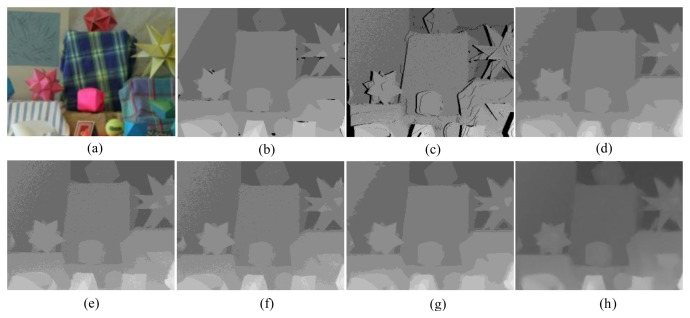
Results for Moebius from the Middlebury database: (**a**) Color image; (**b**) Original depth map (right view); (**c**) Noisy image; (**d**) JBF; (**e**) FMI; (**f**) GFMI; (**g**) GFMIGF; (**h**) Proposed method.

For the quantitative performance evaluation, the test images from Middlebury database [17,18] are used for the ground truth of the synthesized noisy images. That is, depth maps at the left-viewpoint of the stereo pair are rendered to the right-viewpoint using the depth image based rendering (DIBR) [19]. This rendering yields holes around the boundaries of the objects. We then add Kinect-like noise to these rendered depth maps. The noise of Kinect can be modeled by adding white Gaussian noise, together with a deterministic noise that is proportional to the range [20]. The noise we added is similar with [11], which is given as:

(11) $$N(d)=k_{1}d+k_{2}f(d)$$

where *N*(*d*) denotes the noise at the depth value *d, f*(*d*) is a random noise drawn from a zero-mean normal distribution with a variance depending on the depth value, and *k_1_* and *k_2_* are two coefficients with *k_1_* = 0.001, *k_2_* = 2, and the signal-to-noise ratio (SNR) is 25 dB. Figures 15c, 16c, 17c, 18c and 19c illustrate the right-viewpoint depth maps with Kinect like noise using the stereo images from the Middlebury database. The noisy depth maps are filtered and holes are filled by existing methods, and then the results are compared with ground truth right-viewpoint depth maps. The experimental results are shown in Figures 15, 16, 17, 18 and 19. As can be seen, the proposed method outperforms the other methods specifically at the positions near object boundaries. The methods of FMI (Figures 15e, 16e, 17e, 18e and 19e) and GFMI (Figures 15f, 16f, 17f, 18f and 19f) are found to be quite sensitive to the noise pixels. The JBF (Figures 15d, 16d, 17d, 18d and 19d) and GFMIGF (Figures 15g, 16g, 17g, 18g and 19g) perform well but often produce artifacts around depth boundaries. The proposed method (Figures 15h, 16h, 17h, 18h and 19h) avoids such artifacts and produces sharp depth boundaries. For instance, in Figure 18, our proposed method yields clear edges at the boundary of two objects made by the same wood material. Meanwhile the JBF yields blurry boundaries and GFMIGF yields blurry and cluttered boundaries. Table 2 shows the PSNR of the compared methods for the quantitative evaluation. The proposed method yields the highest PSNR. The PSNR gain of the proposed method is about 1 dB in average for all tested images than the second-best GFMIGF method.

**Figure 20.**
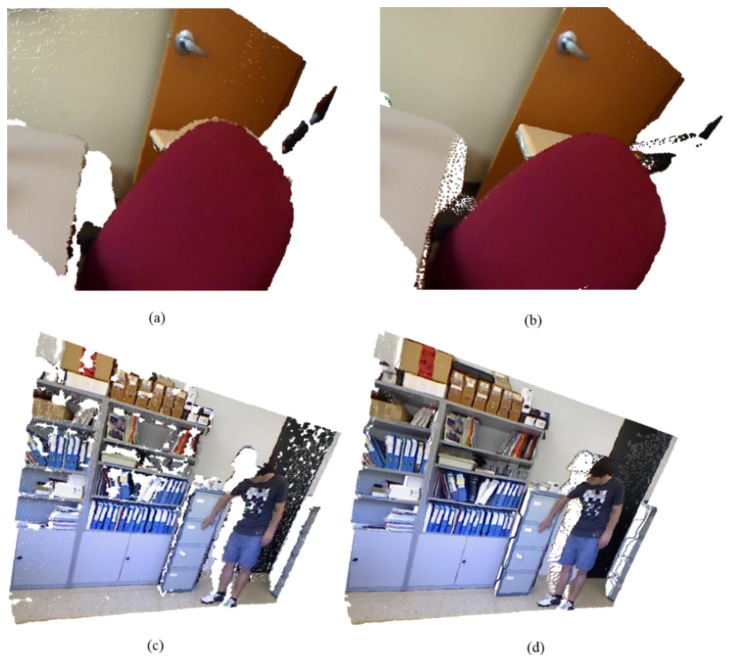
3-D rendered color images using: (**a**,**c**) raw depth map; (**b**,**d**) the depth map obtained by the proposed method.

To illustrate the impact of the quality improvement achieved by the proposed method, we use the filtered depth map obtained by the proposed algorithm to represent a color image in three-dimensional (3-D) perspective as shown in Figure 20. The high-quality rendered image indicates that the proposed method can be applied to 3-D displays. More specifically, a DIBR technique using the depth map obtained by the proposed method is expected to render higher quality 3-D images.

## 4. Conclusions

A novel way of exploiting the edge information for the depth map enhancement is proposed. We allow the local support of the window for the filtering to vary adaptively according to the direction of the edge and the relative position between the edge extracted from the color image and the to-be-filtered pixel as well. Our filtering approach is implemented for the hole filling problem of the Kinect depth images. The proposed method showed that using the adaptive directional filter kernel with adaptive filter range gives better hole filling results especially for the hole pixels near object boundaries. The effectiveness of the proposed method was demonstrated quantitatively by using the synthetic test images and qualitatively using the Kinect test images.

## Figures and Tables

**Table 1.** Used filters in the proposed method.

	**Non-Edge**	**Edge**
Non-Hole	Joint Trilateral Filter (JTF) [13]	Directional Joint Bilateral Filter (DJBF)
Hole	Partial Directional Joint Bilateral Filter (PDJBF)	Directional Joint Bilateral Filter (DJBF)

**Table 2.** PSNR comparisons.

	**JTF**	**FMI**	**GFMI**	**GFMIGF**	**Proposed**
Lampshade	32.10	27.69	28.02	32.99	34.11
Plastic	32.41	30.66	30.79	32.81	33.82
Venus	40.02	34.40	34.73	40.32	41.13
Wood	28.95	22.10	23.59	28.54	30.79
Moebius	37.08	33.31	33.62	37.42	37.71
Average	34.11	29.63	30.15	34.42	35.51
